# Contrast-enhanced ultrasonography to evaluate changes in renal cortical microcirculation induced by noradrenaline: a pilot study

**DOI:** 10.1186/s13054-014-0653-3

**Published:** 2014-12-02

**Authors:** Antoine G Schneider, Mark D Goodwin, Anthony Schelleman, Michael Bailey, Lynne Johnson, Rinaldo Bellomo

**Affiliations:** Intensive Care Unit, Austin Health, 145 Studley road, Heidelberg, VIC 3084 Australia; Radiology department, Austin Health, Heidelberg, VIC Australia; Australian and New Zealand Intensive Care Research Centre, School of Public Health and Preventive Medicine, Monash University, Melbourne, VIC Australia; Faculty of Medicine, University of Melbourne, Melbourne, VIC Australia

## Abstract

**Introduction:**

We used contrast-enhanced ultrasound (CEUS) to estimate the effect of an increase in mean arterial pressure (MAP) induced by noradrenaline infusion on renal microvascular cortical perfusion in critically ill patients.

**Methods:**

Twelve patients requiring a noradrenaline infusion to maintain a MAP more than 60 mmHg within 48 hours of intensive care unit admission were included in the study. Renal CEUS scans with destruction-replenishment sequences and Sonovue® (Bracco, Milano Italy) as a contrast agent, were performed at baseline (MAP 60 to 65 mmHg) and after a noradrenaline-induced increase in MAP to 80 to 85 mmHg.

**Results:**

There was no adverse effect associated with ultrasound contrast agent administration or increase in noradrenaline infusion rate. Adequate images were obtained in all patients at all study times. To reach the higher MAP target, median noradrenaline infusion rate was increased from 10 to 14 μg/min.

Noradrenaline-induced increases in MAP were not associated with a significant change in overall CEUS derived mean perfusion indices (median perfusion index 3056 (interquartile range: 2438 to 6771) arbitrary units (a.u.) at baseline versus 4101 (3067 to 5981) a.u. after MAP increase, *P* = 0.38). At individual level, however, we observed important heterogeneity in responses (range -51% to +97% changes from baseline).

**Conclusions:**

A noradrenaline-induced increase in MAP was not associated with an overall increase in renal cortical perfusion as estimated by CEUS. However, at individual level, such response was heterogeneous and unpredictable suggesting great variability in pressure responsiveness within a cohort with a similar clinical phenotype.

**Electronic supplementary material:**

The online version of this article (doi:10.1186/s13054-014-0653-3) contains supplementary material, which is available to authorized users.

## Introduction

Renal blood flow (RBF) and glomerular filtration rate (GFR) are normally autoregulated when systemic mean arterial pressure (MAP) is maintained between 60 and 100 mmHg [1]. This autoregulation is thought [2,3] to be mediated by a fast myogenic response of the afferent arteriole to blood pressure changes [4] with a superimposed slower tubuloglomerular feedback mechanism [5]. In the setting of acute kidney injury (AKI), however, as demonstrated in several animal models [6-9] this autoregulation may be lost and further episodes of hypotension may be associated with marked decreases in renal blood flow (RBF) and glomerular filtration rate (GFR). Similarly, in patients with chronic hypertension, the autoregulatory curve is shifted to the right [10], and a higher MAP is required to maintain RBF and GFR.

Based on this knowledge, intensive care physicians often aim at maintaining a higher MAP in patients deemed at risk of AKI and in those with chronic hypertension [11]. An arbitrary target of 70 or 80 mmHg is usually chosen and therapy adapted to reach this goal. Vasoconstrictors such as noradrenaline often need to be started or their infusion rate increased.

However, such an increase in the MAP target means higher exposure to a drug with recognized dose-dependent side effects [12]. To date, there are no data confirming that such practice improves renal microcirculation. In addition, microcirculatory changes in response to an increase in MAP might depend on individual circumstances and a standardized MAP target might not suit every patient [13]. Hence, a technique that allows microvascular renal perfusion quantification at the patient-level might help clinicians to determine the optimal MAP in critically ill patients.

Contrast-enhanced ultrasonography (CEUS) is a novel imaging technique that uses low mechanical-index ultrasonography and microbubble-based contrast agents. CEUS has been shown to be able to detect changes in microvascular RBF [14-17]. CEUS is fast, safe, has good inter-observer agreement [18] and can be performed at the bedside without requiring patient transport. It is therefore an ideal technique to be used in the ICU [18].

We have designed a pilot observational study using CEUS to determine changes in renal cortical microvascular blood flow associated with noradrenaline-induced increases in MAP from 60 to 65 mmHg to 80 to 85 mmHg in critically ill patients and related these changes to clinical outcomes.

## Methods

The study was approved by the Austin Health Research Ethics Committee (H2012/04592).

### Participant recruitment and selection

Twelve patients who had an arterial line *in situ*, required a noradrenaline infusion >5 μg/minute and expected to require noradrenaline for >24 hours at the time of inclusion, were approached and consented within 48 hours of ICU admission (consent could be obtained from the patient or next of kin). Exclusion criteria were: Sonovue® or any ultrasound contrast agent (UCA) intolerance, intra-cranial hypertension, aortic dissection or aneurysm, decompensated heart failure, severe left ventricular dysfunction (left ventricular ejection fraction <30%), ischaemic heart disease, ventricular arrhythmia, end-stage renal disease (pre-morbid plasma creatinine concentration >300 μmol/l or chronic haemodialysis), ongoing renal replacement therapy (RRT), inability to obtain informed consent, concern of the treating physician that a MAP of 60 mmHg might be too low or a MAP of 80 mmHg might be too high, and enrolment in a conflicting research study.

### Study procedure

After obtaining written consent, renal CEUS scans were performed (procedure detailed below). The first scan was performed at baseline with the commonly applied MAP level of 60 to 65 mmHg. The noradrenaline infusion was then titrated up to reach a MAP of 80 to 85 mmHg. After a 30-minute equilibration period, the CEUS scan was repeated. The rate of the noradrenaline infusion was then titrated back to the original MAP target as per treating physician recommendations.

### Safety parameters

All studies were performed within the ICU. Patients received full monitoring according to their clinical stability at the time of the examination as evaluated by the treating physician. At a minimum, invasive blood pressure, blood oxygen saturation (via pulse oxymetry), and continuous three-lead electrocardiograms were available in all patients throughout the experiment. In addition, urinary output was monitored on an hourly basis and blood samples regularly drawn for routine blood tests as per clinical practice.

### CEUS procedure

For the purpose of this study, we used Sonovue® (Bracco, Milano, Italy) as an ultrasound contrast agent (UCA). The UCA was infused into a peripheral or central vein (according to availability) through an intravenous cannula using a dedicated syringe pump. Low mechanical index ultrasound of the kidney was performed with a Philips IU22® ultrasound machine and a C5-1® 5 MHz probe. A long-axis view of the kidney was obtained by placing the transducer probe over the lower back of the subject. Once adequate images of the kidney were obtained, UCA infusion was started at 1 ml/minute. Image depth, focus, gain and frame rate were optimized at the beginning of each experiment and held constant during the study. After a 2-minute period required to obtain a steady state, five consecutive destruction/refilling sequences (with 15-s refilling time) were obtained [19]. Destruction was obtained by applying a flash of increased ultrasound intensity (five pulses with high mechanical index (>1.0).

### Sequence analyses

**Figure 1:**
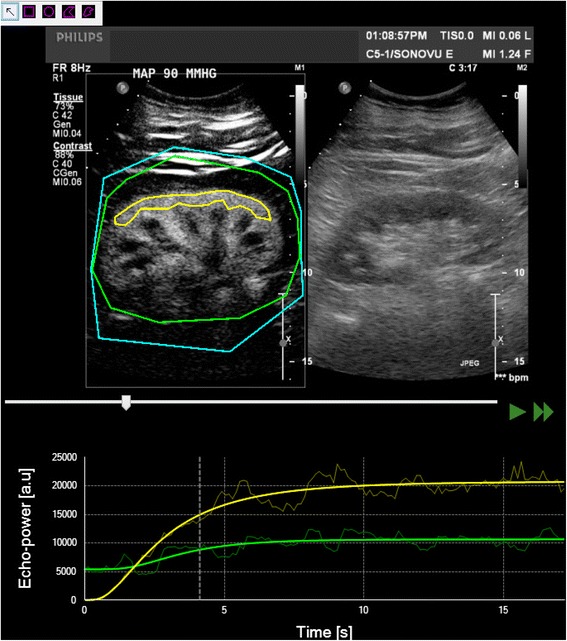
**Sequence analysis with Vuebox®.** A region of interest was drawn (yellow line) in the largest possible area of renal cortex close to the ultrasound probe. The software generates a time intensity curve (in yellow in lower panel). This curve is used to generate contrast-enhanced ultrasound (CEUS)-derived parameters. The green curve corresponds to the overall zone (kidney and surrounding tissues) and is not relevant for analysis. T0 corresponds to the destruction of all microbubbles in the scan plan by an ultrasound flash (increase of ultrasound intensity see text for details). Top right panel shows standard B-mode ultrasound image.

Ultrasound datasets were exported in a digital imaging and communication in medicine (DICOM) format and analysed offline using a dedicated software package, VueBox® (Bracco Research, Geneva, Switzerland). An example of offline analysis is presented in Figure 1. For each sequence, one region of interest (ROI) was drawn. In order to minimize the influence of local perfusion heterogeneities, this ROI was drawn so that it enclosed the largest area of visible renal cortex on the surface of the kidney closest to the ultrasound probe. Cortex that was only intermittently visible because of breathing or other artefacts was not included in the ROI. The software generates a perfusion index (PI), which is thought to be proportional to perfusion within a region of interest. The PI is calculated by dividing the relative blood volume (RBV) by the mean transit time (mTT) and is expressed in arbitrary units (a.u.). These parameters have been described in detail elsewhere [19,20]. In brief, the RBV is a measure of pixel luminance. RBV is proportional to contrast agent concentration within a ROI (increases with higher level of perfusion) and is expressed in a.u. The mTT is a measure of the time to replenishment after flash destruction of the contrast agent (a shorter time indicates higher level of perfusion). MTT is expressed in seconds.

Suboptimal sequences with inadequate insonification or excessive movement artefact were excluded. For each patient and study time, the median value for interpretable measurements was considered for analysis. Given the expected inter-observation variability and based on previous research [17], a change of more than 25% between two measurements was considered to be significant.

### Consistency of CEUS measurements

As perfusion indices are calculated based on two measured parameters (RBV and mTT), we sought to report the agreement between these two measurements. For the purpose of this analysis, we considered that changes in RBV and changes in mTT were consistent when, in a given patient, both parameters increased or decreased by >25% of their baseline value in a direction indicating similar change in blood flow (for instance a >25% increase in RBV and a >25% decrease in mTT, both indicating an increase in perfusion), or when one parameter increased or decreased by >25% of its baseline value and the other one was unchanged (<25% change). We considered that changes were *not consistent* when both parameters increased or decreased by >25% of their baseline value in a direction indicating an opposite change in blood flow. The value of 25% corresponds to the mean coefficient of variation for mTT.

### Responders versus non responders

Patients were classified into responders if their PI increased by >25% after noradrenaline-induced increase in MAP compared with baseline. They were classified into non responders if PI increased by <25% or decreased after a noradrenaline-induced increase in MAP.

### Statistical analysis

Analyses were performed using SPSS® version 21 (IBM, Armonk, NY, USA). All outcomes were assessed for normality and as RBV, mTT and PI were all well-approximated by log-normal distributions, each was log-transformed prior to analysis. Precision of measurements was estimated by calculating the coefficient of variation, defined as SD divided by the mean value and multiplied by 100 for all sequences obtained at one study time. We report the mean average coefficient of variation for the three CEUS-derived parameters. Normally distributed data are reported as mean (SD) and compared using the paired *t*-test. Non-normally distributed data are presented as median (interquartile range) and were compared using Wilcoxon matched-pairs signed-rank test. A two-sided *P*-value of 0.05 was considered to be statistically significant.

## Results

### Patients’ description and outcomes

Patients; demographics and outcomes are detailed in Table 1. Additional data on patients’ haemodynamic, sedation and ventilation status at the time of CEUS studies are presented in Additional file 1, available online. Septic shock was the main diagnosis for ten of the twelve patients; six were mechanically ventilated at the time of the study.

**Table 1:** **Patients’ characteristics and outcomes**

	**On ICU admission**						**At the time of CEUS study**							**Outcomes**			
**Patient number**	**Age, years**	**Main diagnosis**	**Chronic kidney disease**	**Diabetes**	**Hypertension**	**APACHE-III score**	**Mechanical ventilation**	**RIFLE**	**NA dosage (μg/minute)**	**Time on NA (hrs)**	**Other inotropes**	**ACE Inh < 48 h**	**Diuretics < 48 hrs**	**ICU LOS, days**	**RIFLE**	**RRT**	**Death**
1	51	Septic shock	No	No	No	65	Yes	R	13	46	Milrinone	No	No	6	F	No	No
2	64	Septic shock	No	Yes	No	47	No	-	6	20	No	Yes	Yes	5	R	No	No
3	30	Septic shock	No	No	No	18	Yes	-	3.5	6	No	No	No	4	I	No	No
4	71	Septic shock	No	No	No	57	No	R	4	26	No	No	No	2	I	No	No
5	84	Septic shock	No	No	Yes	73	Yes	R	14	52	No	No	Yes	10	R	No	Yes
6	42	Status epilepticus	No	No	No	24	Yes	R	10	48	No	No	Yes	12	R	No	No
7	68	Septic shock	No	Yes	Yes	60	No	-	2	17	No	No	No	3	-	No	No
8	63	Septic shock	No	No	No	61	Yes	I	15	19	No	No	No	4	I	No	No
9	32	Septic shock	No	No	No	56	No	-	11	5	No	No	No	2	R	No	No
10	71	Septic shock	No	No	Yes	44	No	F	10	21	No	No	No	2	F	No	Yes
11	62	Septic shock	No	No	No	80	No	I	16	8	Vasopressin	No	No	7	I	No	No
12	65	Cardiogenic shock	No	No	No	75	Yes	-	8	16	No	No	Yes	4	-	No	No

APACHE, acute physiology and chronic health evaluation; NA, noradrenaline; RIFLE, risk, injury, failure, loss, end-stage kidney failure; ACE Inh, angiotensin converting enzyme inhibitors; LOS, length of stay; RRT, renal replacement therapy.

Ten patients developed AKI during their hospital stay according to the RIFLE classification [21] but none required RRT and all had recovered their renal function at the time of hospital discharge or death. Two patients died during their hospital stay.

### Tolerance/feasibility

Overall, 24 CEUS scans were performed using a total of 48 vials of Sonovue® (10 ml per scan). No adverse effect was noted with UCA administration. At least one interpretable sequence was obtained for each patient at the two study times. Each CEUS examination took approximately 15 minutes to complete. To increase MAP from 60 to 65 mmHg to 80 to 85 Hg, the median noradrenaline infusion rate was increased from 10 μg/minute (interquartile range (IQR) 5.5 to 12.5) to 14 μg/minute (IQR 10.5 to 18.5). No adverse event was associated with the increase in MAP and noradrenaline dose.

### Precision

The mean coefficients of variation were 12.2% for RBV, 25.2% for mTT and 25.2% for PI.

### CEUS-derived parameters

#### Perfusion indices (PI)

**Figure 2:**
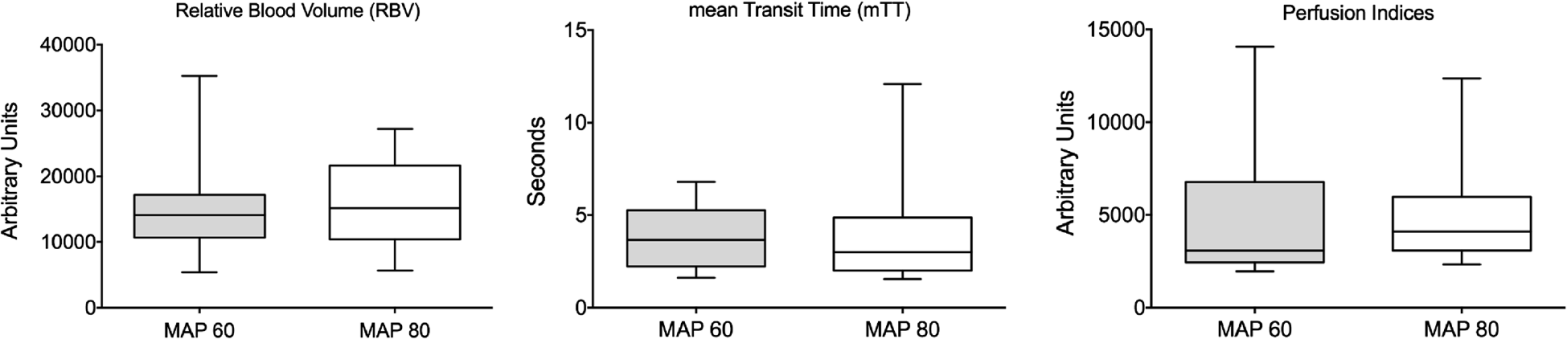
**Overall results.** MAP, mean arterial pressure.

Overall (Figure 2), there was no difference in perfusion indices (PI) between measurements obtained at baseline (median PI 3056 (2,438 to 6,771) a.u.) and those obtained after a noradrenaline-induced increase in MAP (4101 (3067 to 5981) a.u.); *P* = 0.38.

**Figure 3:**
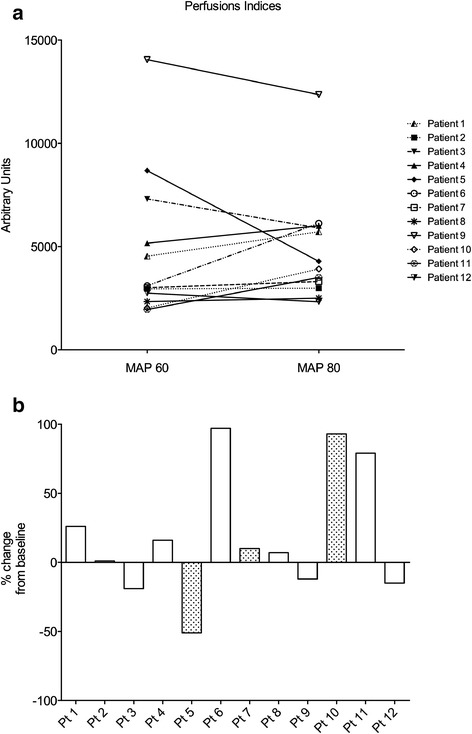
**Perfusion indices patient per patient. (a)** Raw data (arbitrary units). **(b)** Percentage change from baseline. Grey bars are for patients (Pt) with chronic hypertension (Pt 5, 7 and 10). MAP, mean arterial pressure.

However, at the individual level (Figure 3) large variations were observed. Indeed, >25% increase was observed in four patients (>75% in three) and >25% decrease was observed in one patient. Smaller changes were observed in the seven remaining patients (−19 to +16%).

Among the three patients with chronic hypertension, noradrenaline-induced MAP increase was associated with 10% and 93% increase in two patients, but 50% decrease in the remaining patient.

#### Consistency of changes in parameters

**Figure 4:**
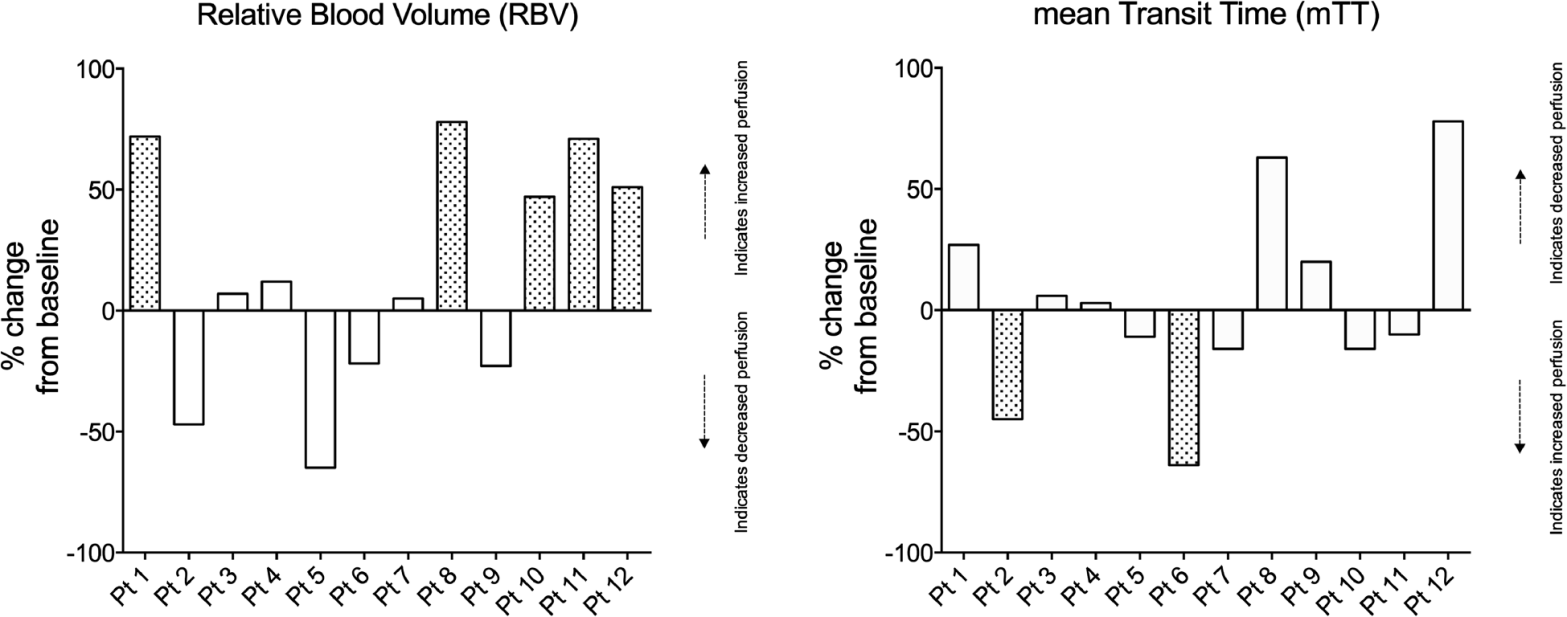
**Percentage changes from baseline for relative blood volume (RBV) and mean transit time (mTT) parameters.** Agreement between the two parameters obtained to determine perfusion indices. An increase in mTT is suggestive of a decrease in organ perfusion while a increase in RBV is suggestive of an increase in organ perfusion. Changes in RBV and changes in mTT were considered to be consistent when, in a given patient, both parameters increased or decreased by >25% of their baseline value in a direction indicating similar change in blood flow or when one parameter increased or decreased by >25% of its baseline value and the other one was unchanged (<25% change). Grey bars are for patients with chronic hypertension (patients (Pt) 5, 7 and 10).

Patient-level changes from baseline for RBV and mTT are presented in Figure 4. Such changes were considered to be consistent with each other (suggesting similar changes in perfusion) in nine (75%) patients. They were not consistent (suggesting opposite changes in perfusion) in three patients (Table 1, patients 2, 8 and 12).

#### Relationship between CEUS parameters and clinical outcomes

Four patients (patients 1, 6, 10 and 11 in Table 1) were classified as responders based on a >25% change in PI after noradrenaline-induced increase in MAP. These patients had an average APACHE III score of 56.3 and one had hypertension. All four patients developed AKI (RIFLE-F in two patients, RIFLE-I in one patient and RIFLE-R in one patient) and one subsequently died. Conversely, within the eight patients who were classified as non-responders, the average APACHE III score was 55.9, two had diabetes mellitus (DM) and one hypertension (HT). Six (75%) developed AKI (RIFLE-R in three and RIFLE-I in three patients).

## Discussion

### Key findings

We attempted to quantify renal cortical microvascular perfusion in a non-invasive manner in critically ill patients on vasopressors using CEUS. We found that renal CEUS was feasible and administration of UCA well-tolerated even in haemodynamically unstable patients. We found that a noradrenaline-induced increase in MAP was not associated with an overall change in renal perfusion indices as measured by CEUS. In contrast, the intervention was associated with highly heterogeneous responses at a patient-level, with observed increase or decrease by >25% of baseline values in a quarter of the patients.

### Relationship to previous studies

Several animal studies [22,23] have demonstrated that noradrenaline may increase RBF in vasodilated/hypotensive states. This effect seems to be mediated by an increase in systemic blood pressure and an associated decrease in renal sympathetic tone through a baroreceptor response [23]. The effect of noradrenaline *per se* on renal vasculature tone was examined in an animal model of septic shock [24]. In this study, although noradrenaline administration was associated with an increase in MAP under all conditions, it was only associated with an increase in RBF (as measured by implanted ultrasonic flowmeters) when renal vascular vasodilatation was present. These findings suggest that noradrenaline infusion, in acute endotoxemia reverses systemic hypotension and may improve RBF independent of perfusion pressure.

However, the human data confirming these experimental findings is extremely limited. There are only a few studies that report RBF measurement in critical illness and its changes in response to noradrenaline administration. In particular, Redfors *et al*. [25] have measured global RBF in critically ill patients, with invasive renal vein blood sampling. In this very detailed physiological study, an increase from 60 to 75 mmHg of the MAP was associated with an increase in GFR and urine flow but not in RBF.

Other authors have used surrogate measures of RBF and measured renal vascular resistive indices in critically ill patients. These indices, however, have been shown to be poorly correlated with RBF [26]. However, such parameters can be predictive of reversibility of AKI [27] and perform better than urinary indices for diagnosing persistent AKI.

Current recommendations for MAP target [11] in septic shock (grade 1C) are based on small physiological studies that demonstrated the absence of changes in several physiological parameters [28,29]. A recent large clinical trial [12] randomly allocated 776 patients with septic shock to undergo resuscitation with a MAP target of either 65 to 70 mmHg or 80 to 85 mmHg. In this trial, there was no difference in 28- or 90-day mortality between the two groups. In the subgroup of patients with chronic hypertension, however, there was a decrease in the need for RRT. Our data, suggesting high heterogeneity in renal perfusion in response to a similar change in MAP, could provide an explanation for these findings. Indeed, a pre-determined one-size-fits-all MAP target might not be suitable for a highly heterogeneous group such as critically ill patients. On the contrary, a tailored MAP target aiming at restoring tissue perfusion, based on assessment of mental status, skin appearance, urinary output and perhaps CEUS parameters could represent an alternative approach [30].

### Strengths and limitations

This study is the first to use CEUS to evaluate renal microvascular perfusion induced by a change in the noradrenaline infusion rate. CEUS is a new technology, which is applicable at the bedside and could improve our understanding of organ perfusion in critical illness. This study provides pathophysiological insight into an important and unresolved question that persists despite large randomized controlled trials. However, this study has several limitations. First, the small sample size precludes advanced statistical analyses and determination of factors predicting response and the classification of patients into responders and non-responders remains arbitrary. Second, no measure of renal vascular resistive indices was performed. If consistent with CEUS data, this measure would have made our conclusions stronger. However, for technical reasons, such data were not collected.

Changes between measurements could be random variations associated with an overly sensitive technique. Indeed, CEUS measurements can be limited by numerous factors such as organ depth, echogenicity of surrounding tissues, breathing artefacts, US equipment settings, and fluid retention. This is illustrated by the large variability of baseline measurements among patients. However, such parameters are unlikely to have influenced the results because, for each patient, both CEUS scans were performed within a very short time window (<45 minutes) in which ventilation parameters, fluid and medications infusions, patient-position and US machine settings were all kept constant. Only comparison of measurements obtained in a single patient, as all other factors are kept constant can be interpreted. CEUS data were obtained by a single experienced operator aware of all these limitations.

In ventilated patients with low tidal volumes, respiration-related renal displacement can be dealt with by selecting a probe angle limiting this motion and by the use of an advanced image stabilisation algorithm in the VueBox® software. Therefore, a breath-holding manoeuvre was not necessary.

Consistency between parameters used to determine perfusion indices was fairly good, however, 25% of measurements suggested changes in perfusion in opposite directions. Further studies would be required to clarify the causes of such disagreements, how to prevent them and how to handle them.

Finally, the clinical relevance of our findings and the applicability of CEUS derived parameters remains to be determined. Indeed, as illustrated by a recent animal study [31] the relationship between renal microcirculation and renal function are complex and still poorly understood. Our findings suggest the need for further studies aiming at understanding factors that predict changes in CEUS-derived parameters and to evaluate whether the presence or absence of change in CEUS-derived parameters in response to an increase in MAP are associated with specific clinical outcomes.

## Conclusions

An increase in MAP as induced by noradrenaline infusion was not associated with overall changes in renal microvascular cortical perfusion as evaluated by CEUS. However, some individual patients seem to have marked responses (either increase or decrease). Further studies are required to establish whether such patients would benefit from tailored MAP targets.

## Key messages

- A noradrenaline-induced increase in MAP is not associated with an overall increase in renal cortical perfusion as estimated by CEUS
- However, at individual level, such response was heterogeneous and unpredictable, suggesting great variability in pressure responsiveness within a cohort with a similar clinical phenotype

## Additional file

Additional file 1:
**Patients’ hemodynamic/sedation/ventilation status at the time of contrast-enhanced ultrasound CEUS studies.**

## Abbreviations

AKI: acute kidney injury
APACHE: acute physiology and chronic health evaluation
CEUS: contrast-enhanced ultrasound
DICOM: digital imaging and communication in medicine
GFR: glomerular filtration rate
IQR: interquartile range
MAP: mean arterial pressure
MI: mechanical index
mTT: mean transit time
PI: perfusion index
RBF: renal blood flow
RBV: Relative blood volume
RIFLE: risk, injury, failure, loss, end-stage renal failure
ROI: region of interest
RRT: renal replacement therapy
UCA: ultrasound contrast agent
