# Supplementary material for: Contrast-enhanced ultrasonography to evaluate changes in renal cortical microcirculation induced by noradrenaline: a pilot study
Source: Crit Care. 2014 Dec 2;18(6):653. doi: 10.1186/s13054-014-0653-3 (PMC4262130; doi:10.1186/s13054-014-0653-3)
Supplement: Additional file 1: — Patients’ hemodynamic/sedation/ventilation status at the time of contrast-enhanced ultrasound CEUS studies. [file 13054_2014_653_MOESM1_ESM.doc]

**Additional file 1. Patients’ hemodynamic / sedation / ventilation status at the time of CEUS studies**

| **Patient Number** | **Central venous pressure (mmHg)** | **Heart Rate (bpm)** | **Cardiac index (L/min/m^2^)** | **Heart Rhythm** | **Sedation regimen** | **PEEP** | **PaCO2** | **pH** | **Serum lactate level (mmol/l)** |
| --- | --- | --- | --- | --- | --- | --- | --- | --- | --- |
| 1 | 13 | 85 | 2.6 | SR | Propofol / Fentanyl | 12 | 39 | 7.39 | 2.7 |
| 2 | 8 | 70 | NA | PPM | - | - | 35 | 7.42 | 1.4 |
| 3 | 3 | 85 | NA | SR | Propofol / Fentanyl | 8 | 48 | 7.38 | 2.2 |
| 4 | 5 | 105 | NA | ST | - | - | 48 | 7.38 | 2.3 |
| 5 | 17 | 130 | 2.7 | AF | Propofol / Morphine | 5 | 40 | 7.45 | 1.2 |
| 6 | 8 | 90 | NA | SR | Thiopental / Midazolam | 5 | 35 | 7.51 | 1.3 |
| 7 | 13 | 70 | NA | SR | - | - | 40 | 7.45 | 1.3 |
| 8 | 9 | 105 | NA | ST | Propofol / Fentanyl | 5 | 40 | 7.44 | 2.2 |
| 9 | 8 | 80 | NA | SR | - | - | 32 | 7.49 | 1.4 |
| 10 | 10 | 100 | NA | SR | - | - | 42 | 7.31 | 1.6 |
| 11 | 10 | 95 | 2.2 | SR | - | - | 26 | 7.47 | 7.6 |
| 12 | 8 | 70 | 2.6 | SR | Propofol / Morphine | 8 | 33 | 7.42 | 1.9 |
|  |  |  |  |  |  |  |  |  |  |
|  |  |  |  |  |  |  |  |  |  |
|  |  |  |  |  |  |  |  |  |  |

Bpm= beats per minute

SR: Sinus rhythm

PPM: Permanent pacemaker

ST: sinus tachycardia

PEEP: Positive end expiratory Pressure
